# Influence of Dance Programmes on Gait Parameters and Physical Parameters of the Lower Body in Older People: A Systematic Review

**DOI:** 10.3390/ijerph19031547

**Published:** 2022-01-29

**Authors:** Bárbara Rodríguez, Federico Paris-Garcia

**Affiliations:** Section of Physical Education and Sports, Department of Sports and Computer Science, Faculty of Sports Sciences, University Pablo de Olavide, ES-41013 Seville, Spain; barbararodriguez639@gmail.com

**Keywords:** systematic review, dance intervention, ageing, gait, mobility

## Abstract

(1) Background: The regular practice of dancing benefits the physical condition, improving quality of life and minimising the adverse effects of ageing. Therefore, this review aims to evaluate the impact of dance programmes by quantifying different physical parameters of the lower body in older adults. (2) Methods: A systematic qualitative review in the English language (PubMed, Scopus, OvidSP, Cochrane and PEDro database) until mid-2020 considering the PRISMA guidelines and the PEDro quality criteria considering the following parameters of gait: stride length and width, speed. Physical parameters: flexion and dorsiflexion joint, muscle strength and range of motion were carried out. (3) Results: 9 studies with a population of 544 subjects from 5 continents and 6 types of dances were taken into account. The improvement of some parameters over others depended on the type of dance and the movements generated, having moderate positive effects on strength, agility, mobility and balance. (4) Conclusions: there is a general improvement in the functional capacity of the elderly through the practice of ballroom dancing, with specific improvement of each parameter depending on the type of dance.

## 1. Introduction

According to the National Institute of Statistics (INE), this year (2021), 19.77% of the Spanish population are over 64 years of age [1]. In 2010, the percentage was 17%, so the population has aged by 2.33% in the last eleven years. In addition, the dependency rate of those over 64 years of age has also increased from 24.89% in 2010 to 30.46% in 2021, the latter being the highest figure in the country’s history [2]. These data are in line with global data where 12% of the population is over 65 years of age (2018 data) and is expected to reach 1.5 billion by 2050 [3].

Human ageing is a natural and gradual process in which functional, neurochemical, morphological and psychological changes occur, resulting in a loss of functional capacity of the individual [4]. During this involutional process, strength, muscle mass, flexibility and coordination decrease. In addition, there is a progressive visual loss and deficiencies in visual and spatial skills and decreased mobility and postural balance, affecting executive functions among other capacities [4,5]. For this reason, the physical performance and vitality of the elderly are declining, thus, increasing their dependency and vulnerability [5,6,7].

Postural control is a multifactorial mechanism formed by the sensory systems: visual, proprioceptive and vestibular. Even if one fails, postural balance is maintained [5]. In addition, other factors, such as the strength of the lower extremities, are essential to maintain postural control [8]. Furthermore, the ankle joint and its flexibility play a crucial role in body balance, supporting the body’s weight [9]. There is evidence that muscle strength in the plantar and dorsal flexion of the ankle, along with poor postural control and balance, is associated with a high risk of falls in older adults [10].

Other authors argue that the force in the plantar flexor muscles benefits static balance because the soleus is the first muscle to be activated in the forward swing [11]. This evidence shows the importance of the strength of the muscles related to dorsiflexion and plantar flexion of the ankle in static equilibrium.

Furthermore, slower gait speed at these ages is associated with mobility-related fatigue due to a lack of muscle strength [12,13]. Ageing affects the central nervous system and the neuromuscular system, contributing to gait and balance performance [9]. There is accumulating evidence that dance practice improves gait speed [14,15], balance and strength [15,16].

Currently, there are many physical activity programmes to improve these parameters to counteract the effects of ageing. Still, an alternative that is becoming increasingly popular in this age group is dance programmes. Dance requires physical activity accompanied by music and involves social interaction when practised as a couple or in a group [17]. In addition, this is considered an attractive activity which is key to obtaining the benefits of its practical adherence [16].

On one hand, dance combines aerobic fitness, sensorimotor skills and adaptivity, making the risk of injury low [17]. On the other hand, dance requires the audiovisual integration of visual, sensory and auditory stimuli [18]. Together with this, dance carries aesthetic connotations, which promotes the self-motivation of the subject and greater involvement in it, improving not only motor capacity but also the psycho-social and behavioural sphere [19,20]. Through dance, the individual plans, controls and executes a sequence of actions, which benefits cognitive health and executive function [8].

In this sense, previous systematic reviews have analysed the effect of dance programmes on multiple parameters related to health, such as cognitive function [21], metabolic parameters [22], Parkinson’s disease [23], the prevention of falls [24] and functional capacity [21].

Taking as a reference the last systematic review [21], which focused on functional capacity, currently, there are recent proposals that focus on physio-mechanical aspects of the lower body that evaluate the effect of dance on parameters not contemplated in that review, such as ankle muscle strength in plantar flexion and dorsiflexion, as well as in knee extension, walking ability, stride length, the speed of weight transfer, turning time and balance in daily tasks.

The purpose of this study is to carry out an updated qualitative systematic review based on the PRISMA guidelines (Supplement S1) [25], taking into account the PEDro quality criteria to analyse the effect of different dance programmes on physical and mechanical parameters of the lower body linked to gait [26].

## 2. Materials and Methods

### 2.1. Information Sources and Search Strategy

According to the criteria established by the PRISMA guidelines, the present review was carried out and took into account the quality criteria for studies in the health field according to the PEDro database (Spanish version of the scale of PEDro quality criteria).

In order to establish a strategy to develop the framework, an extensive review of the bibliography in different databases (PubMed, Scopus, OvidSP, Cochrane and PEDro database) was consulted. In the first phase (identification process), without a time limit, several concepts that would establish the basis of the conceptual framework in the search engine of the previous databases were entered. All studies published from their inception to the search date were considered.

The study search process was conducted by a single researcher (BR) and discussion with FPG. The search strategies and results in each database are described in Supplement S2.

The descriptors used in the previous phase were: dance (Dance), mobility (Mobility), and the elderly (Elderly) combined with the Boolean operators AND, that is: “Dance AND Mobility AND Elderly”.

### 2.2. Eligibility Criteria

The inclusion criteria for this review were as follows: (1) Population: Healthy elderly people over 60 without pathologies. (2) Intervention: studies with an exclusive dance programme intervention without other variables. (3) Outcome: Parameter values related to mobility, gait and physical-mechanical parameters of the lower limbs were considered. In particular, lower body muscle strength values (ankle muscle strength with the FTSST test, plantar flexion LP-ROM and RP-ROM, knee extension through the SPPB test) have been taken into account. Linked to gait were four variables: gait speed, walking ability (velocity), length and width of stride. Related to balance were the following parameters: dynamic balance, static balance, postural control, functional balance of daily tasks, the centre of pressure (COP) and, finally, weight balance. (4) Study design: clinical studies, regardless of the type, such as randomised controlled clinical trials (RCTs), non-randomised controlled clinical trials and before–after studies. (5) Language: Only publications in English were considered. The exclusion criteria were duplicated studies, abstracts, reviews, descriptive studies, systematic review studies based on the description of a protocol, studies carried out before 2000 and studies based on the point of view of the authors.

### 2.3. Study Selection

All documents retrieved from the study search process were imported into Mendeley Desktop (version 1.19.1). After removing duplicates, the titles and abstracts of papers were examined for inclusion. After the initial selection, the texts of the remaining papers were carefully reviewed and assessed for final inclusion. Two independent investigators carried out the study search process (FPG and BR). Any discrepancies between the two researchers were discussed and were resolved by consensus.

### 2.4. List of Acronyms Used

The following table displays the acronyms used for the main parameters considered for the review to synthesise information in the following tables (see Table 1).

### 2.5. Data Extraction

A pre-defined table of data in Excel 2016 (Microsoft, Redmond, WA, USA) by two independent researchers (BR and FPG) was used for the data extraction process.

The following items were taken into account from the included studies: publication year, first author’s name, country of publication, sample size and withdrawals, details of participants, treatment and control intervention, duration of intervention, outcome measures and results. In particular, details of the intervention dance programme and parameters of the lower part of the body and gait parameters were extracted. Any disagreements between the two researchers were discussed and resolved through consensus.

### 2.6. Characteristics of the Sample of Studies Considered

Data considered for the discussion section of this review were extracted after an exhaustive reading of the full text. The following items were selected as relevant in the research: authors and publication of the article, objective, participants, type of article and details of the intervention, measures of the results and conclusions.

The main characteristics of the selected articles were the following:

- Year of publication: two studies belong to 2021, another two studies from 2019, another two from 2018, one from 2017, one from 2015 and another one from 2012. Therefore, seven have been published in the last five years;
- Language: all articles are in English;
- Country: two studies have taken place in Thailand, one in China, one in Denmark, one in Italy, one in the USA, one in Brazil, one in Portugal and another one in Switzerland. A broad geographic dispersion encompassing different cultures can be observed;
- Dance style applied in the programmes: four articles used traditional dance (Sriachiangmai, chain dance from the Faroe Islands, Thai dance and traditional Brazilian dances “el Forró” and “Sertanejo”), one adapted tap dance, one combined ballroom dancing, line dancing and grabbing dances from the beginning of the 19th century, one focused on line dancing, one on creative dance and one on salsa (Latin rhythms).

### 2.7. Characteristics of the Sample

Taking into account the nine selected articles, the participants had the following characteristics:

- Age range: ranged between 60 and 93 years, with the range from 60–75 being the most frequent age, with the participation of older adults over 80 years of age lower;
- Gender: six of the nine studies included people of both sexes as participants, while in three of them only women. There was a higher percentage of participation of women than men in all studies;
- Health of the subjects (inclusion criteria in each studies considered): one subject free of limitations for participation in exercise and who had not had any falls in the last year; one subject without dementia with the ability to maintain activity for 30 min; one able to walk without walkers or canes without the presence of uncontrolled cardiovascular disease, diabetes, stroke, severe osteoarthritis or significant musculoskeletal pain in the lower extremities or back in the past six months; one retiree, living independently, without medical conditions (acute or chronic illness or motor deficit); one with the ability to understand instructions with no use of ambulatory assistive devices, no neurological disorders, no use of portable oxygen and no internal cardiac defibrillator or myocardial infarction in the previous six months; one subject independent in all daily activities with no recent history of bone fracture or surgery; one subject partially or self-sufficient in daily living tasks without the use of an assistive device, without cognitive impairment, with the absence of cardiovascular, neuromuscular or neurological disorders and does not take medications that may affect the programme; one subject with no history of musculoskeletal, neurological, or orthopedic disorders, able to walk independently without an assistive device and without prior experience.;
- Level of physical activity: in seven articles, the subjects were independent in the routines of daily life and were not enroled in any sporting activity or participants in physical activity, one was a sedentary subject, one was physically active.

### 2.8. Parameters Evaluated

The variables considered in the articles are a part of the present review (see Table 2). These articles also addressed other secondary variables such as psycho-social aspects and other physiological aspects related to body composition (weight, height, muscle mass and body fat), cardiovascular level, heart rate (HR) at rest and blood pressure (BP) (see Table 2).

Regarding mobility, the distribution of the main parameters through the articles considered were (for more detail see Table 2): six studies evaluated gait speed, five studies evaluated walking ability and two evaluated stride length.

The following variables (force parameters) were only addressed in one article: the strength of the muscles in plantar flexion and dorsiflexion of the ankle (study 1 [10]). Study 6 [27] addressed the strength of the knee flexors and extensors. Study 2 [28] analysed the strength of the upper extremities (see Table 2).

Aspects related to balance are addressed in different articles. Studies 3 [29], 7 [30] and 9 [31] analysed static equilibrium. Five studies [10,30,31,32,33] analysed dynamic balance. Studies 2, 3 and 9 [28,29,31] analysed postural control. Studies 4, 6 and 7 [27,30,32] analysed daily tasks in relation to functional capacity. Finally, only study 4 [32] analysed weight bearing and study 7 [30] analysed the centre of pressure (COP) (see Table 2).

Other aspects related to mobility linked to aspects of the lower body were evaluated in the sample studies. Five studies [27,28,32,33,34] evaluated agility. Study 1 [10] analysed the flexion–extension of the knee and study 8 [33] analysed the parameter of the range of motion of the joint. Six studies [10,27,28,29,33,34] evaluated different physiological parameters (previously detailed) related to the health status of the participants (see Table 3).

### 2.9. Publication Bias

Assessment of publication bias using a funnel plot was planned in the protocol [35]; however, the assessment was not conducted, as there were no more than ten studies included in each meta-analysis.

### 2.10. Risk of Bias Assessment and Methodological Quality

Risk of bias was evaluated by evaluating the methodological quality of each randomised clinical trial (RCT) using the Physiotherapy Evidence Database (PEDro) scale [26]. PEDro scores ranged from 2 to 9 points, with a mean score for the set of samples of 8 points (Table 4). All of the selected studies scored 7 or more, indicating the high quality of the selected trials. All of the studies specified the eligibility criteria. In all studies except one (study 5 [34]), the subjects were randomly allocated to groups. All of them showed similarities at the baseline (studies 1–9).

In three studies (studies 1, 4 and 5) [10,32,34], the trials had blinded participants or therapists, and three had blinded assessors (studies 1, 4 and 8) [10,32,33]. Seven trials had retention rates of 85% or greater (studies 1, 3, 5–9) [10,27,29,30,31,33,34], and all of the studies met the intention-to-treat analysis criteria (studies 1–9) [10,27,28,29,30,31,32,33,34]. All of the studies applied statistical analysis to group differences (studies 1–9) [10,27,28,29,30,31,32,33,34] and reported point estimates and measurements of variability (studies 1–9) [10,27,28,29,30,31,32,33,34] as well. No studies were excluded on the basis of their methodological quality.

## 3. Results

### 3.1. Study Selection

The number of records for each database consulted was: PubMed (103), Scopus (33), OvidSP (557), Cochrane (17) and PEDro (10). The initial number of articles considered can be seen from the terms used for all the databases consulted (720 articles). The sample was reduced to 307 as 413 records were identified as duplicated and, therefore, eliminated. No additional records as other sources were identified. 

Once all the records considered for the identification phase were obtained, new inclusion/exclusion criteria were applied. The characteristics of the sample (>60 years) were considered. For this, all those studies whose subjects had a registered pathology were discarded. The type of intervention programme and its effect on the physical parameters of the lower body (muscle strength) or biomechanical variables related to gait such as balance or mobility (screening phase) were considered. This phase excluded 283 records, leaving 24 records applying the abovementioned criteria.

After reading all the selected studies carefully, for different reasons, fifteen studies were discarded: reviews (six registries), multicomponent interventions (eight registries) and others (one registry, No available text). Finally, the set of articles considered for the review was 9.

**Figure 1:**
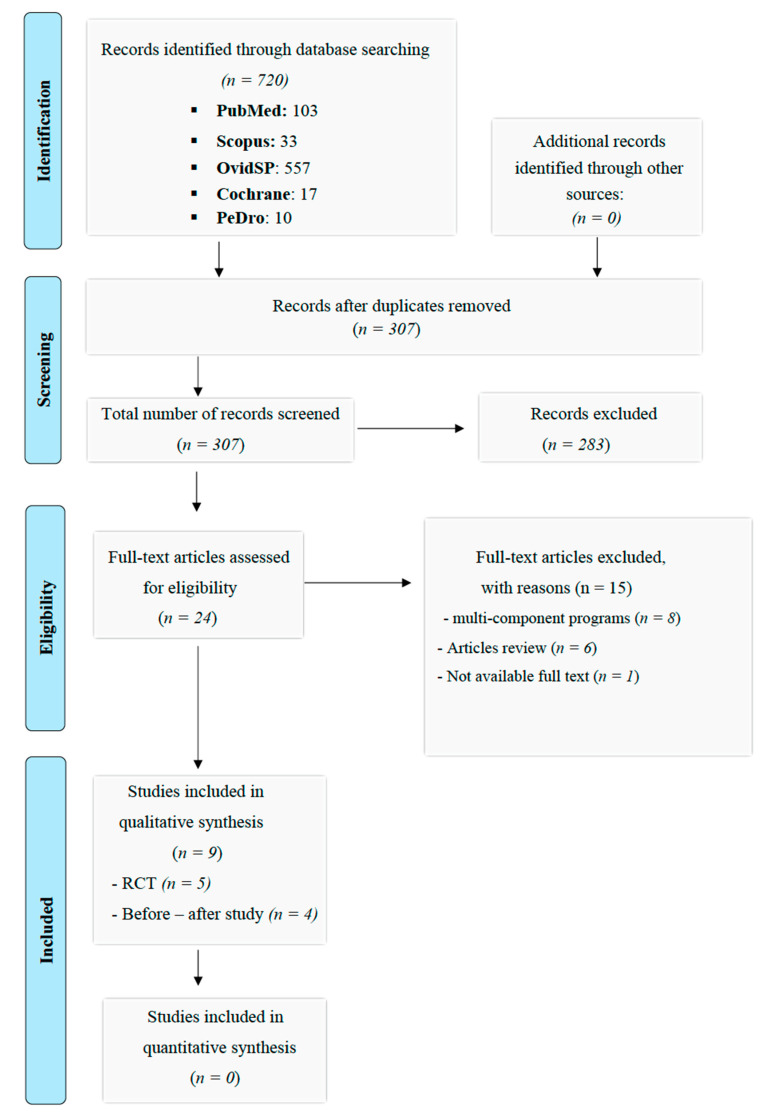
Flow diagram.

The following flow chart shows the methodology to select the sample of articles considered for the present review (see Figure 1). This process of review was conducted in three phases: identification phase, a screening phase and, finally, an eligibility phase was carried out.

### 3.2. Muscular Force

Six articles evaluated the variable of muscular strength, as the strength of the lower extremities are the most treated (five studies) and the one of interest for the discussion of the results (see Table 5).

In Study 1 [10], it was evaluated whether an adapted tap programme could lead to improvements in ankle muscle strength. The intervention group showed higher improvements than the control group at six weeks of intervention, in ankle muscle strength (with the FTSST test) and in plantar flexion (LP-ROM and RP-ROM).

In this sense, in study 6 [27], relevant results in the force generated by knee extension were obtained. However, there were no differences in knee flexion strength. The results of the SPPB test obtained higher values regarding the function of the lower extremities. However, study 9 [33] also evaluated the power of the leg extensors, finding no significant differences (*p* > 0.05) after eight weeks of intervention.

In study 8 [33], the greatest increases in strength values were obtained with respect to the rest of the study variables, with reaching 21%, being a significant improvement (*p* < 0.05).

In study 2 [28], lower body and upper body muscle strength were addressed, both of which improved after training.

### 3.3. Parameters Linked to the Gait

Six articles evaluated gait using three variables: gait speed, walking ability and stride length, which is discussed below (see Table 5).

Study 2 [28] showed positive results in the ability to walk and gait times improved being faster after the intervention. These effects were also evidenced in study 3 [29] where the dance group improved performance in the gait test, the ability to walk and the speed of gait with no apparent changes in the control group.

Study 4 [32] also found significant improvements (*p* < 0.05) in walking ability and gait speed, as in previous trials. The peculiarity of the results of this study was an increase in the length and width of the stride.

In Study 6 [27], the intervention group significantly improved gait speed (*p* < 0.05). However, this improvement was not significant (*p* > 0.05) compared to the difficulty in walking 400 m.

In study 7 [30], improvements in gait and gait speed were obtained, thus, improving functional performance.

Finally, the salsa dance programme for eight weeks, carried out in study 9 [31], presented significant increases (*p* < 0.05) in the speed, length and time of the stride, while there were no significant increases (*p* > 0.05) in the control group. However, the application of the intervention programme had no effect on various measures related to gait variability.

### 3.4. Balance

Seven articles dealt with balance, the following variables being in the order of frequency of appearance: dynamic balance, static balance and postural control, functional balance or daily tasks, the centre of pressure (COP) and, finally, weight balance (see Table 5).

Dynamic balance was significantly higher (*p* < 0.05) in the dance group in Study 2 [28]. In this sense, dynamic equilibrium improved by 13% in study 8 [33]. Study 9 [31], evidenced an improvement in static postural control, particularly dynamic balance.

In study 3 [29], the results of the two tests (BBS and FAB) that evaluated postural balance found significant improvements (*p* < 0.05) in the intervention group compared to the control group.

In study 7 [30], positive increases of several variables related to balance in the intervention group were obtained, while no significant differences in the control group (*p* < 0.05) were found. The test scores (TUG and TT) were higher, showing improvement in the functional performance of the subjects. Static balance was enhanced in the length of the trajectory of the COP, the oscillation speed and the dynamic balance.

Study 4 [32] obtained improvements in balance in general (functional, static and dynamic), the speed of the weight swing was lower and there was a faster weight transfer.

Improvements in functional balance in Study 6 [27] were also found. By obtaining better results on the BBS test and improving confidence, the older adults in the intervention group sample had lower perceived mobility limitations. In the functional balance of daily tasks, the self-reported limitation on the difficulty of climbing stairs was reduced.

### 3.5. Other Outcomes Related to Mobility: Flexibility and Agility

Agility was addressed in five articles, while flexibility was addressed in one. In study 2 [28], the intervention group obtained a shorter movement time and greater agility and mobility in contrast to the control group (see Table 5). In addition, study 4 [32] found an improvement in agility due to a decrease in the time in the turning movements resulting in faster movement times.

In study 5 [34], execution times in different tests that evaluated agility were considerably reduced, producing a reduction in the execution time of 9.84% in the TUG test, 9.12% in the FSS test and 8.14% in the TUGM test, respectively. In particular, they showed improvements in dual-task skills of 6.58% (physical components) and 5.75 (cognitive components).

The intervention group of study 6 [27] showed positive results related to agility, although no improvement was shown in the difficulty of walking 400 m, the self-reported limitation on the difficulty of climbing stairs was reduced.

Covering the same variables related to the lower body as in the rest of the studies, study 8 [33] focused on others, such as the flexibility of the lower extremities, which improved by 13%. In addition, the same percentage was obtained for the variable of motor agility.

## 4. Discussion

The purpose of this systematic review was to analyse the possible positive effects of dance programmes on mobility and other physio-mechanical variables of the lower body in older adults.

To achieve this objective, a qualitative systematic review was carried out in several databases. Considering the PEDro scale scores for the selected articles, they were attributed an internal validity suitable for comparing and treating their results in this systematic review.

The importance of analysing the muscle strength of the knee on the dominant side is due to its relationship with mobility-related tasks (study 1 [10]). Regarding the knee joint, studies 6 and 9 [27,31] analysed the muscle strength of the knee extensor and flexor after the dance intervention. In the results of study 6 [27], after eight weeks of line dance training, an increase in muscle strength was evidenced in knee extensions. However, no findings in flexion strength were found. The coincidence in these results occurred in another trial where a traditional Korean dance programme was applied for twelve weeks [13]. In study case 9 [31], the participants had a high level of physical activity, more significant than that corresponding to their age, which justifies the lack of findings on the power of the leg extensors.

As can be seen, in the linear dance and Korean traditional dance programmes, muscle strength increases in knee extensions and no findings were found in flexion strength. However, muscle strength does not increase in knee extensions in the salsa programme. The difference between these two programs is the level of previous physical activity of the participants, which seems to be relevant for the achievement of benefits with regards to the muscle strength of the knee extensors.

Regarding the strength in the plantar flexion and ankle dorsiflexion muscles, study 1 [10], after six weeks of an adapted tap programme, found significant changes in the muscular strength of the ankle muscles (FTSST) and the ROM in plantar flexion of the left and right foot. In this sense, the dorsiflexor muscles of the ankle play an essential role in standing on one limb. Other authors [36] associated the improvement in static balance with the gain in strength in the dorsiflexor muscles of the ankle.

In a previous study, lower extremity joint ROM was analysed through a low-impact dance programme for sixteen weeks [37]. The study was carried out with 32 healthy older women randomly assigned to the low-impact dance group (LOD) or to the sedentary group (SED). Significant improvements (*p* < 0.05) were obtained in ankle dorsiflexion, while there were no differences in plantar flexion in post-intervention measurements. Therefore, in study 1 [10], the benefits were obtained in plantar flexion, while in Wu et al. [37], were obtained in ankle dorsiflexion. This difference, relative to the articulation, is due to the type of dance applied. Study 1 [10] focused on adapted tap dancing requiring repetitive ankle joint movements requiring greater work from the lower extremities, while low-impact dance involves global movements integrating all parts of the body, joints and muscle groups [37].

Therefore, in the analysis of the influence of force on the lower body muscles on various parameters of the ankle joint, through intervention with different dances, it can be observed that the dance styles with greater use of specific muscle groups and movements, like repetitive like tap-dancing, help to improve these parameters. However, low-impact programmes associated with gains in ROM in ankle dorsiflexion do not necessarily imply improvements in ankle dorsiflexion and plantar flexion.

In study 2 [28], the intervention group, after twelve weeks practising Srichiangmai traditional dance, showed significant improvement (*p* < 0.05) in muscle strength of the lower extremities. Janyacharoen et al. [38] applied a similar programme of traditional Thai dance for six weeks to women with an average age of 65.8 ± 5.1 years. Using the FTSST, they measured the strength of the lower extremities and participants obtaining faster times (10.2 ± 1.5 versus 14.4 ± 3.3 s). This variable was also significant in an RCT where a traditional Korean dance programme was applied for twelve weeks in older women [13]. Furthermore, in study 8 [33], after a twenty-four-week programme of creative dance, the participants in the intervention group obtained benefits of up to 21% in the lower extremities.

During ageing, there are reductions in the lower extremities’ mass, strength and muscle power. In previous RCTs, the effect of an eight-week dance programme on the architecture of the muscles of the lower extremities of older adult women was analysed, finding a significant decrease in the effect of ageing regarding these parameters [39].

As observed in the bibliography, different dance styles produce significant improvements in the strength of the lower extremities, even causing a modification in the architecture of the muscles. Considering programmes discussed above have a duration of six, twelve and twenty-four weeks, it is shown that from the sixth week, there are improvements in the muscular strength of the lower extremities, regardless of the type of dance. Korean traditional dance, Thai traditional dance and creative dance programmes effectively improve the muscular strength of the lower extremities. However, more studies are required on the rest of the disciplines and shorter application times.

Studies 2–4 [28,29,40] obtained significant results concerning the ability to walk, leading to a reduction in times, with the speed of the march being higher. Previous research (study 7 [30]) has shown improvements in gait and gait speed, thus, improving functional performance. Another work has also shown an increase in gait speed (study 6 [27]). Furthermore, Krampe et al. [12] refer to a higher speed in the dance group than the control group after six weeks (three sessions/week) of applying a dance programme based on the Lebed method applied in adults from 64 to 96 years old. This method is based on choreographies performed with music and low-impact steps.

According to the studies reviewed, it is possible to establish a positive relationship between dance programmes (even low-impact ones) and improvement in walking ability, gait speed and gait in older adults and the elderly from six weeks of duration. Dance programmes, including the styles ballroom dancing, line dancing, traditional Thai dance, Srichiangmai dance, Faroese chain dance and the Lebel method, improve gait and walking ability. The great diversity of these dance styles suggests that any dance could benefit walking ability, leg agility and gait speed.

Study 4 [32] analysed gait and stride, showing an increase in length and width (distance between the midpoints of both heels). In this sense, a significant increase in time, speed and stride length (*p* < 0.05) was obtained in Study 9 [31]. Based on the salsa programme, the latter did not affect the gait variability, being justified in the discussion as logical in salsa practice due to its intrinsic characteristics. In the RCT by Krampe et al. [14] and Jeon et al. [13], there was an increase in stride length in the intervention group at the end of the programme. In both studies [13], and also study 4 [32] (selected in this review), an improvement in the deviation of the trajectory and the turns when walking [13] were obtained. otherwise, the time in the turning movements decreased, resulting in times of faster movements.

Once again, relevance is established as to the type of dance and its characteristics. Salsa improved stride length and speed but did not induce changes in gait variability, while Thai dance, Korean dance and the low-impact Lebed method induced significant benefits in gait agility and the movement of turns.

Several studies in the review showed changes after the dance intervention in dynamic balance, such as studies 2 and 8 [28,33], where it improved by 13% (study 8 [33]) and study 9 [33] showed an improvement in static postural control, particularly dynamic balance. Furthermore, studies 4 and 7 [30,32] alluded to the positive static and dynamic equilibrium effects.

These results are consistent with previous studies after applying a Korean dance programme for twelve weeks (three sessions/week) at 60–70% HRmax adjusted for age in women from 65–75 years of age [13]. The results of standing on one leg and dynamic balance were significantly higher (*p* < 0.05) in the Korean traditional dance group than CG.

Several studies showed improvements in the balance after dance practice; for example, Federici et al. [40], after three months of intervention in a Latin dance program (2 sessions/week) in older adults between 56 and 68 years in contrast to the control group. In study 4 [32], functional balance improvements were obtained. Rodacki et al. [30] showed improved functional performance in two tests (TUG and Tinetti Test). Improvements in functional balance in daily tasks were also obtained in Study 6 [27].

On the one hand, the self-perceived limitation on the difficulty of climbing stairs was reduced. In this sense, other randomised trials provide improved results regarding postural balance [28]. Additionally, subjects over 60 years old who practised social dance performed better on postural stability than other participants aged from 50 to 60 years [29].

On the other hand, in the systematic review by Fong et al. [41], after analysing the effectiveness different styles of dance in 28 programmes, no significant evidence (*p* > 0.05) was found in self-perceived mobility between the dance group and other forms of physical exercise.

Taking into account the characteristics of the subject samples from previous studies, balance improved most significantly in adults older than 60 years of age. In all the studies analysed, there was an improvement in some type of balance. The styles that improved dynamic and static balance were Korean dance, traditional Srichiangmai, creative dance and salsa. In addition, Latin dance, Thai dance and ballroom and line dancing improved functional balance, line dancing improved balance related to daily tasks and, lastly, chain dance and social dance improved postural stability.

After the dance programme application in studies 4 and 7 [30,32], improvements in the speed of weight displacement were obtained, producing a faster transfer. This decrease in time in the movement of the COP also occurred in the study by Sofianidis et al. [36] in the Greek traditional dance intervention group. Furthermore, Kattenstroth et al. [42] also obtained benefits over the COP, giving greater forward shifts. Furthermore, the backward displacements of the COP increased in the lateral direction in the dance group. This implies changing the POPs without falling or taking a step forward, essential in postural stability and prevention of falls.

Studies 2, 4, 5, 6 and 8 [27,28,33,34,40] of the present review analysed agility, coinciding with the improvement obtained with the application of dance programmes, either in the reduction in time of movement or in the execution of more agile movements. Zhang et al. [43], comparing a group of older adults who practised social dance and a control group, showed faster reaction times were obtained in the lower extremities in the dance group.

In order to analyse the effects on personal autonomy and balance, a previous study carried out a ballroom dance programme applied to 75 institutionalised older adults randomly divided into a control group and a dance group. In this study, improvements in functional autonomy were reported, showing a significant reduction (*p* < 0.05) in the execution times of all tests [5].

In another intervention, after applying a dance programme for twelve weeks in older adults, the mean frailty scores decreased by 0.69 at six weeks and 1.06 at twelve weeks. In addition, slowness and weakness gradually decreased in the group dance compared to the control group [44]. In this way, frailty is reduced, and movement agility, essential for daily tasks and executive function, increases.

Another aspect to be treated as a variable related to mobility is flexibility in the lower extremities. Study 8 [33] shows better results after the creative dance intervention. This variable is also measured in the RCT of Janyachaoen et al. [38], where it improved significantly compared to the control group (14.9 ± 3.5 versus 11.1 ± 5.7 cm) (*p* < 0.05).

The agility and flexibility of the lower extremities are crucial factors in preventing falls in older adults and the elderly [8]. Therefore, these findings are essential for the investigation. As for the COP, individual dance, ballroom and line dancing, traditional Thai dance, and Greek dance help transfer weight more quickly, providing postural stability. This improvement in these parameters allows changing the COP movements without falling or stepping forward, thus, avoiding falls [42].

All the studies that analysed agility showed improvements in this variable regardless of the dance practised. As was established above, agility is crucial in the autonomy of the elderly. The dance could help reduce the risk of falling in their performance of daily tasks. In this sense, it could be observed that slowness and fragility decrease from the sixth week, obtaining better results at the twelfth week. This requires further studies since knowing if these effects are increasing as the practice progresses, and if they are maintained over time, would be helpful and relevant to the success of these programmes. According to the two studies reviewed in this discussion, creative and Thai dance improve lower extremity flexibility.

These two types of dance, due to the slow transfer of weight, could maintain the stretching of the musculature for a longer time, improving its length. However, stretching at the end of the class could provide more significant benefits.

Improvements in the physiological parameters have accompanied the results obtained for the physio-mechanical parameters of the lower body. Although not all the selected articles evaluated these items, and it is not the object of study of this review, it is important to emphasise their improvement.

## 5. Conclusions

After an exhaustive review of the different dance programmes and their relationship with the different physical parameters linked to the lower body and gait parameters, the suitability of this scope to improve the functional capacity of this population can be established. Depending on what aspect of functional capacity (balance, mobility, muscular strength or other aspects) is targeted for improvement, a dance programme is more or less recommended over others.

Beyond the results analysed and discussed, there is an increase in proposals to improve the quality of life of the elderly through dance programmes based on improvement in functional capacity.

Adherence is an aspect that is very present in these studies, being higher in this type of proposal compared to other approaches based on physical activity or sports training programmes.

Therefore, the use of dance to improve the quality of life for people through functional capacity is still a valid alternative. Still, future methodologically rigorous research is required to quantify the relationship between the effect of these programmes and specific parameters related to health.

## Figures and Tables

**Table 1:** Main acronyms used.

Parameter	Acronyms
Five Time Sit to Stand Test	(FTSST)
bioelectric impedance analysis	(BIA)
Functional Reach Test	(FRT)
Timed Up and Go Test	(TUG)
Timed Up and Go Dual Task	(TUGM)
6-minute walk test	(6MWT)
Tinetti Test Score	(TT)
Berg Balance Scale	(BBS)
Fullerton Advanced Balance	(FAB)
Short Physical Performance Battery	(SPPB)
Step-Quick-Time	(SQT)
Step Up Over	(SO)
Walk Across	(WA)
Sit to Stand	(SS)
Four Square Step	(FSS)
6-Item Lubben Social Media Scale	(LSNS-650)
Borg Rating of Perceived Exertion Scale	(RPE)
Fried Preclinical Disability Screening	(PCD)
Senior Fitness Test	(SFT)
satisfaction with life scale	(SWLS)
clock drawing test	(Test-CDT)
Mini-Mental State Examination	(MMSE)
Range Of Motion	(ROM)
Center of Pressure	(COP)
Randomized clinical trial	(RCT)
Experimental group	(EG)
Control Group	(CG)

**Table 2:** Variables related to the physical functional capacity and mobility considered in studies reviewed.

Study (Number)	1	2	3	4	5	6	7	8	9	Studies
Authors (Year of Publications)	Wang, Q., & Zhao, Y. (2021)	Buransri, M., & Phanpheng, Y. (2021)	Hofgaard, J., et al. (2019)	Noopud, P., et al. (2019)	Brustio, P. R., et al. (2018)	Bennett, C. G., & Hackney, M. E. (2017)	Rodacki, A. L. F. et al. (2017)	Cruz-Ferreira, A. et al. (2015)	Granacher, U., et al. (2012)	
FORCE PARAMETERS										
Dorsiflexion and plantar flexion of ankle joint	✓									1
Flexion and extension of knee joint						✓				1
Superior limbs		✓								1
Lower limbs		✓				✓		✓	✓	4
WALKING/GAIT										
Velocity of the gait		✓	✓	✓		✓	✓		✓	6
Walking capacity		✓	✓	✓		✓			✓	5
Length of the stride				✓					✓	2
BALANCE										
Static balance				✓			✓		✓	3
Dynamic balance		✓		✓			✓	✓	✓	5
Control postural			✓	✓					✓	3
Daily/functional homeworks				✓		✓	✓			3
Weight balancing				✓						1
COP							✓			1

**Table 3:** Other variables related to mobility and secondary aspects of the psycho-social field were evaluated in the included articles.

Study (Number)	1	2	3	4	5	6	7	8	9	Studies
OTHER ASPECTS OF MOBILITY										
Range of Movement (ROM)								✓		1
Flexion and extension of knee joint	✓									1
Agility		✓		✓	✓	✓		✓		5
Physiological parameters	✓	✓	✓		✓	✓		✓		6
PSYCHOSOCIAL PARAMETERS										
Quality of life					✓					1
Social commitment					✓					1
Satisfaction life								✓		1
Adherence	✓			✓	✓	✓		✓	✓	6

**Table 4:** PEDro scale rating.

Study (Number)	1	2	3	4	5	6	7	8	9
1. Eligibility Criteria	✓	✓	✓	✓	✓	✓	✓	✓	✓
2. Random Allocation	✓	✓	✓	✓		✓	✓	✓	✓
3. Concealed Allocation	✓	✓						✓	✓
4. Group similiar at baseline	✓	✓	✓	✓	✓	✓	✓	✓	✓
5. Blinded subjects	✓			✓	✓				
6. Blinded theparist	✓			✓					
7. Blinded assesors	✓			✓				✓	
8. Less than 15% dropouts	✓		✓		✓	✓	✓	✓	✓
9. Intention-to-treat analysis	✓	✓	✓	✓	✓	✓	✓	✓	✓
10. Between-group comparisons	✓	✓	✓	✓	✓	✓	✓	✓	✓
11. Point measure and variability	✓	✓	✓	✓	✓	✓	✓	✓	✓
PEDro Score	11	7	7	9	7	7	7	9	8

**Table 5:** Parameters evaluated in the studies considered for review.

Authors	Sample	Setting	Intervention Characteristics/Outcomes	Finding
Reference		Length of Intervention		
**1. Wang, Q., & Zhao, Y.** (International Journal of Environmental Research and Public Health 2021;18(12))	n = 44 (9 m, 35 w); EG (22) and CG (22); Mean = 64.1 years	(RCT) 6 weeks 3/week 60–90 min/session 10’ min of warm-up	Parameters: Force, FSST, ROM of Ankle joint and force of plantar flexor muscles	Improvements in FSST, and ROM of experimental group to Control group (both feets). Improvements in large trainnings. Group better results in COP and total walking distance.
			Adherence: 88.3%	Inconsistent results in the postural control of the experimental group
**2. Buransri, M., & Phanpheng, Y.** (Muscles, Ligaments and Tendons Journal 2021; 11(2):215–222)	n = 90; EG (45) and CG (45); Mean = 60–75 years	(RCT) 12 weeks 3/week 45 min/session 5’ min of warm-up streching: 15’ Intensity: 60–75% FCmáx.	Health parameters: blood pressure, FC, weigh, IMC and Body composition (BIA). Equilibrium, movility and FRT (TUG), Walking capacity (6MWT), Force of lower limbs (SS) an Force ol upper limbs	EG and CG improved data from physiological parameters. Significant improves of Equilibrium and mobility. Gait velocity improved past intervervention.
				The intervention improved strength, lower body endurance, and core stability, being the balance and effectiveness of sensitive muscle structures and control of body movement, a primary ability to perform everyday tasks with confidence in advanced ages.
**3. Hofgaard, J., et al.** (Hofgaard, J.; Ermidis, G.; Mohr, M. Biomed Res. Int. 2019, 9)	n = 25 (9 m,16 w); EG (15) and CG (10); Mean ± SD = 75 ± 5 years	(RCT) 6 weeks 2/week Session 1–6 of 30 min/session. The rest session 45 min/session	1 week between measurements. Health parameters: BP, resting HR, muscle mass and body fat content. Postural balance: BBS and FAB. Mobility: SPPB, TUG, 6MWT, 30 sec sitting and standing test.	The BP was reduced more than in the CG, the BBS and FAB scores improved, the latter being higher than the CG, in the 6-min walk, the 30-second sitting and standing test, and TUG improved only in the IG and body fat content was reduced in GI, with no change in CG.
				6 weeks of Faroese chain dance training had beneficial effects, significantly improving postural balance, physical function and overall health.
**4. Noopud, P., et al.** (Aging Clinical and Experimental Research 2019; 31(7): 961–967)	n = 43 (43 m); EG (22) and CG (21); Mean = 60–80 years	(RCT) 12 weeks 3/week 30–60 min/session	2 evaluations (pre/post intervention). Functional Balance (FB): standardized tests of the NeuroCom Balance Master® system. SQT assesses agility and balance, balance and time of movement and WA, walking speed, stride width and length. The TUG test assesses agility. BBS that assesses FB	Improvements in Balance (TTDG) in EG. Significantly lower rocking speed and faster weight transfer in SS test *(p* ≤ 0.001) and TTDG. Faster turning time in SQT (*p* ≤ 0.001), improved SO and WA, with faster movement times, gait speed and a better score on TUG after training (*p* ≤ 0.001).
			Adherence: 88.3%	Thai traditional dance could potentially prevent age-related mobility and balance and related risk of falls.
**5. Brustio, P., et al.** (Geriatric Nursing2018; 39(6): 635-639)	n = 163 (40 m,123w); Mean ± SD = 70 ± 4 years	16 weeks 2/week 60 min/session 10’ warm up. 40’ (slowly waltz, tango and foxtrot, polka, mazurka, and bachata or country) 10’ cooling (breathing exercises).	2 evaluations (pre/post intervention). Movility: TUG, TUGM, FSS.	Improvements (*p* < 0.05) in the mobility of a single task as in that of two tasks. Reduction in 9.84% (TUG), 9.12% (FSS) and 8.14% (TUGM) of t'. Dual task skills improve and 6.58% improve the physical components and 5.75% the mental ones.
			Adherence: 85%	The individual/pair dance has positive effects on the mobility of one or double tasks.
**6. Bennett, C. G., & Hackney, M. E.** (Disability and Rehabilitation 2018; 40 (11): 1259–1265)	n = 23 (3 m, 20w); EG (12) and CG (11); Mean = 65–93 years	(RCT) 8 weeks 2/week 60 min/session 10’ warm up 40’ Main part of session 10’ cooling Intensity: medium	2 evaluations (pre/post intervention) Balance in daily tasks with the BBS. The strength of the knee extensors and knee flexors of the dominant side Lower extremities: SPPB. Gait speed and mobility limitations: 400m walk test. The limitation of perceived mobility: PCD.	The self-reported difficulty of climbing stairs was reduced but not the difficulty of walking 400 m. 8 weeks of line dancing improved knee muscle strength, lower extremity function, gait speed, endurance, and perceived mobility limitations.
			Adherence: 80%	Line dancing involves socializing, which can increase enjoyment and adherence. It involves dynamic control of balance and large muscle groups in the lower extremities to improve physical function and reduce mobility limitations.
**7. Rodacki, A. L. F. et al.** (Topics in Geriatric Rehabilitation 2017; 33 (4): 244–249)	n = 30 (30 w); EG (15) mean ± SD = 69.1 ± 6.6 years and CG (15) mean ± SD = 71.5 ± 7.5 years	(RCT) 8 weeks 3/week 60 min/session 10’ warm up 40’ specific dance (boleros, waltzes and typical Brazilian dances “Forró” and “Sertanejo”) 10’ cooling Intensity: 60–70% FCmáx.	2 evaluations (pre/post intervention) Functional performance: 6MWT, TT and TUG; length of COP, the mean oscillation speed, the area of oscillation of HR and the dynamic equilibrium with the test of the steps.	Functional performance improved in the Tinetti test, TUG and 6 min walk; the static equilibrium in the path length of the COP, the oscillation speed and the medium frequency oscillation area, and the dynamic equilibrium. CG remained unchanged.
				Ballroom dance-based training is an attractive stimulus for older adults. They improved the static and dynamic conditions of balance and functional performance, thus helping to prevent falls.
**8. Cruz-Ferreira, A. et al.** (Research on Aging 2015; 37(8):837–855)	n = 57 (57w); EG (32) and CG (25); Mean = 65–80 years	(RCT) 24 weeks 3/week 50’/ session 15’ warm-up 25’ main part of session (exercises of balance, agility, strength, flexibility and coordination) 10’ cooling (relaxation and breathing). Creative Dance (CD): they associate images with corporal expression.	Evaluation: middle and final intervention. SFT, 6MWT, flexibility with chair sit-down test, motor agility/dynamic balance through 8-foot rise and fall test, and body composition.	Differences between CE and CG (*p* < 0.05) post-intervention. The GE better physical condition than GC, also improving strength, aerobic endurance, flexibility, motor agility and dynamic balance. Better EC than CG (Friedman Test) (*p* < 0.05) post-intervention.
			Adherence: 85%	21% lower limb strength, 10% aerobic resistance and 13% lower limb flexibility and dynamic balance, 4% weight, 8% waist circumference and 5% BMI. The CG did not show improvement in physical fitness after the intervention.
**9. Granacher, U., et al.** (Gerontology 2012; 58(4): 305–312)	n = 28 (w y m); EG (14) and CG (14); Mean = 63–82 years	(RCT) 8 weeks 2/week 60 min/session 10’ warm-up (static and dynamic balance exercises in salsa), 45’ salsa (individual and in pairs) 5’ cool-down.	2 evaluations (pre/post intervention). CDT and MMSE test. Static postural control by balancing on one leg on a balance platform. Dynamic postural control: walking on a pressure-sensitive instrumentalized walkway. Leg extensor power: countermovement jump on force platform.	The salsa-based intervention program is safe, feasible, and enjoyable for older adults. It improves static postural control, especially the dynamic one, helping prevent falls. More specific training is needed to improve space-time gait variability and muscle power.
			Adherence: 92.5%	Stride speed, length and time improved significantly. It did not affect various measures of gait variability and leg extensor power.

## Data Availability

The data extracted from the studies included and data used for all analyses were all included in this manuscript.

## Supplementary Materials

The following data are available online at https://www.mdpi.com/article/10.3390/ijerph19031547/s1, Supplement S1: PRISMA 2020 Checklist, Supplement S2: Search terms used in each database.
