# Supplementary material for: Influence of Dance Programmes on Gait Parameters and Physical Parameters of the Lower Body in Older People: A Systematic Review
Source: Int J Environ Res Public Health. 2022 Jan 29;19(3):1547. doi: 10.3390/ijerph19031547 (PMC8835694; doi:10.3390/ijerph19031547)
Supplement: Supplementary file 1 [file ijerph-19-01547-s001.zip › Supplement S2.pdf]

Supplement S2. Search terms used in each database

MEDLINE via PubMed

	Searches	Results
#1	dance [MeSH] AND mobility [Title/Abstract] AND elderly [Title/Abstract]	144
#2	dance [MeSH] AND mobility [Title/Abstract] AND elderly [Title/Abstract] Filters applied: in the last 10 years.	101
#3	dance [MeSH] AND mobility [Title/Abstract] AND elderly [Title/Abstract]. Filters applied: in the last 10 years, Aged: 65+ years	62
#4	dance [MeSH] AND mobility [Title/Abstract] AND elderly [Title/Abstract] NOT disease. Filters applied: in the last 10 years, Aged: 65+ years	37

SCOPUS

	Searches	Results
#1	(dance AND mobility AND elderly) AND (LIMIT-TO (PUBYEAR,2021) OR LIMIT-TO (OUBYEAR,2020) OR LIMIT-TO (PUBYEAR,2019) OR LIMIT-TO (PUBYEAR,2018) OR LIMIT-TO (PUBYEAR,2017) OR LIMIT-TO (PUBYEAR,2016) OR LIMIT-TO (PUBYEAR,2015) OR LIMIT-TO (PUBYEAR,2014) OR LIMIT-TO (PUBYEAR,2013) OR LIMIT-TO (PUBYEAR,2012) OR LIMIT-TO (PUBYEAR,2011)	33

OVIDSP

	Searches	Results
#1	dance [MeSH] AND mobility [Title/Abstract] AND elderly [Title/Abstract]	557
#2	dance [MeSH] AND mobility [Title/Abstract] AND elderly [Title/Abstract] NO disease	78

COCHRANE

	Searches	Results
#1	dance [MeSH] AND mobility [Title/Abstract] AND elderly [Title/Abstract]	17

PEDRO

	Searches	Results
#1	dance [MeSH] AND mobility [Title/Abstract] AND elderly [Title/Abstract]	10
